# Supplementary material for: Long live the wasp: adult longevity in captive colonies of the eusocial paper wasp Polistes canadensis (L.)
Source: PeerJ. 2015 Mar 24;3:e848. doi: 10.7717/peerj.848 (PMC4375972; doi:10.7717/peerj.848)
Supplement: Supplementary file 1 [file peerj-03-848-s002.docx]

1

Breslow (Generalized Wilcoxon)	Colony	M2		M3		M4		M5		M6		M7	
		X^2	Sig.	X^2	Sig.	X^2	Sig.	X^2	Sig.	X^2	Sig.	X^2	Sig.
	M1	1.093	.296	1.837	.175	4.058	**.044**	4.693	**.030**	.085	.770	3.795	.051
	M2			.058	.810	3.491	.062	4.325	**.038**	2.691	.101	1.593	.207
	M3					.941	.332	1.773	.183	2.735	.098	.893	.345
	M4							.230	.631	4.713	**.030**	.171	.679
	M5									4.843	**.028**	.473	.492
	M6											5.000	**.025**
