# Supplementary material for: Long live the wasp: adult longevity in captive colonies of the eusocial paper wasp Polistes canadensis (L.)
Source: PeerJ. 2015 Mar 24;3:e848. doi: 10.7717/peerj.848 (PMC4375972; doi:10.7717/peerj.848)
Supplement: Supplementary file 2 [file peerj-03-848-s003.docx]

	Colony	M10B		M8A		M8B		M9A		M9B	
		X^2	Sig.	X^2	Sig.	X^2	Sig.	X^2	Sig.	X^2	Sig.
Breslow (Generalized Wilcoxon)	M10A	.394	.530	2.797	.094	.004	.947	1.225	.268	.214	.643
	M10B			6.880	.009	.459	.498	2.441	.118	1.364	.243
	M8A					3.829	.050	4.340	.037	4.103	.043
	M8B							.832	.362	.280	.597
	M9A									.173	.677
