# Supplementary material for: Long live the wasp: adult longevity in captive colonies of the eusocial paper wasp Polistes canadensis (L.)
Source: PeerJ. 2015 Mar 24;3:e848. doi: 10.7717/peerj.848 (PMC4375972; doi:10.7717/peerj.848)
Supplement: Supplemental Information 4 — Comparison of average lifespans in a selections of ants, bees and all records of wasps, detailing (where possible) the min/max life span and study size. Also detailed is weather studies focused on wild colonies or not. Data collected from peer reviewed articles sourced from https://webofknowledge.com/. [file peerj-03-848-s004.docx]

| Group | *Species* | Caste | condition | Temporal Region | References | Life Span (Days) | Min. Life span (Days) | Max. Lifespan (Days) | Standard Error | Standard Deviation | Total Individuals |
| --- | --- | --- | --- | --- | --- | --- | --- | --- | --- | --- | --- |
| ANT | *Lasius niger* | Q | Unknown | Europe | Jemelity et al 2005 | 10950 | na | na | na | na | na |
| ANT | *Lasius niger* | W | Unknown | Europe | Jemelity et al 2005 | 730 | na | na | na | na | na |
| ANT | *Lasius flavus* | Q | Assisted | UK & Germany | Kutter and Stumper 1969 | 6570 | na | na | na | na | na |
| ANT | *Formica exsecta* | Q | Assisted |  | Pamilo 1991 | 7300 |  |  |  |  |  |
| ANT | *Leptothorax lichtensteini* | Q | Assisted | France | Holldobler and Wilson 1990 | 5475 | na | 5478.63 | na | na | na |
| ANT | *Leptothorax lichtensteini* | W | Assisted | France | Holldobler and Wilson 1990 | 912.5 | na | na | na | na | na |
| ANT | *Atta sexdens* | Q | Assisted |  | Keller 1998 | 4745 | na | na | na | na | na |
| ANT | *Aphaenogaster rudis* | Q | Assisted | USA | Holldobler and Wilson 1990 | 3175.5 | 1680.11 | 4748.15 | na | na | na |
| ANT | *Aphaenogaster rudis* | W | Assisted | USA | Holldobler and Wilson 1990 | 1095 | na | na | na | na | na |
| ANT | *Camponotus consobrinus* | Q | Assisted | Australia | Holldobler and Wilson 1990 | 2555 | na | na | na | na | na |
| ANT | *Myrmecia nigriceps* | W | Assisted | Australia | Haskins and Haskins 1980 | 839.5 | na | 913.11 | na | na | na |
| ANT | *Myrmecia pilosula* | W | Assisted | Australia | Haskins and Haskins 1980 | 474.5 | na | 620.91 | na | na | na |
| ANT | *Solenopsis invicta* | W | Assisted | USA | Porter and Tschinkel 1985 in Holldobler and Wilson 1990 | 280 | na | 679 | na | na | na |
| ANT | *Monomorium pharaonis* | Q | Unknown | ? | Peacock and Baxter 1950 in Holldobler and Wilson 1990 | 273 | na | na | na | na | na |
| ANT | *Monomorium pharaonis* | W | Unknown | ? | Peacock and Baxter 1950 in Holldobler and Wilson 1990 | 70 | na | na | na | na | na |
| BEE | *Apis mellifera* | Q | Assisted |  | Page & Peng 2001 | 1460 | na | 2921.94 | na | na | na |
| BEE | *Apis mellifera* | W | Assisted |  | Page & Peng 2001 | 49 | na | 320 | na | na | na |
| BEE | *Lasioglossum nigripes* | Q | Wild |  | Michener 1969 | 401.5 | na | na | na | na | na |
| BEE | *Lasioglossum nigripes* | W | Wild |  | Michener 1969 | 120 | na | na | na | na | na |
| BEE | *Austroplebeia australis* | W | Wild |  | Halcroft et al 2013 | 161.4 |  |  |  | 6.1 |  |
| BEE | *Bombus fervidus* | W | Assisted | USA | Goldblatt and Fell (1986) | 34.1 | na | na | na | na | na |
| BEE | *Bombus pennsylvanicus* | W | Assisted | USA | Goldblatt and Fell (1986) | 34.1 | na | na | na | na | na |
| BEE | *Bombus atratus* | W | Assisted |  | Vieira da Silva-Matos & Garofalo 2000 | 20.95 |  |  |  |  |  |
| WASP | *Mischocyttarus cerberus styx* | Q | Wild | Rio Claro, Brazil | Giannotti, 1999 | 191.1 | 76 | 448 | na | 99.7 | 17 |
| WASP | *Mischocyttarus cerberus styx* | W | Wild | Rio Claro, Brazil | Giannotti, 1999 | 14 | 2 | 95 | na | 11.6 | 345 |
| WASP | *Mischocyttarus drewseni* | W | Assisted | Lower Amazon, Brazil | Jeanne, 1975 | 61 | 39 | 88 | na | 18.7 | 6 |
| WASP | *Mischocyttarus drewseni* | Q | Assisted | Lower Amazon, Brazil | Jeanne, 1975 | 31 | 6 | 97 | na | 16.9 | 148 |
| WASP | *Polistes lanio* | Q | Wild | Rio Claro, Brazil | Giannotti & Machado, 1994 | 209.15 | ? | ? | ? | ? | ? |
| WASP | *Polistes lanio* | W | Wild | Rio Claro, Brazil | Giannotti & Machado, 1994 | 28.28 | ? | ? | ? | ? | ? |
| WASP | *Polistes canadensis* | adult | Lab | Colon, Panama | This Study | 193 |  | 506 |  | 10.5 | 143 |
| WASP | *Polistes canadensis* | W | Wild | Central-Western Brazil | Torres et al., 2013 | 37.06 | 1 | 126 | 29.07 | 29.07 | na |
| WASP | *Polistes simillimus* | Q | Wild | Rio Claro, Brazil | Giannotti, 2012 | 186 | na | na | na | na | 1 |
| WASP | *Polistes simillimus* |  | Wild | Rio Claro, Brazil | Giannotti, 2012 | 29.1 | 4 | 50 | 12.8 | 12.8 | 360 |
| WASP | *Polistes exclamans* | Q | Wild | Texas, USA | Strassmann, 1985 | 112.5 | na | na | na | na | 27 |
| WASP | *Polistes exclamans* | Q | Wild | Texas, USA | Strassmann, 1985 | 118 | na | na | na | na | 33 |
| WASP | *Polistes exclamans* | W | Wild | Texas, USA | Strassmann, 1985 | 14 | na | 84 | na | 11 | 497 |
| WASP | *Polistes exclamans* | W | Wild | Texas, USA | Strassmann, 1985 | 14 | na | 66 | na | 10 | 222 |
| WASP | *Polistes exclamans* | W | Wild | Texas, USA | Strassmann, 1985 | 17 | na | 102 | na | 14 | 503 |
| WASP | *Polistes dominula* | Q | Wild | Michigan, USA | Gamboa et al., 2002 | 87 | na | na | na | 26 | 13 or lower? |
| WASP | *Polistes fuscatus* | Q | Assisted | Michigan, USA | Gamboa et al., 2002 | 68 | na | na | na | 27 | 13 or lower? |
| WASP | *Polistes fuscatus* | W | Assisted | Michigan, USA | West-Eberhard 1969 | 22.8 | 2 | 30 | na | 10.7 | 32 |
| WASP | *Polistes cinerascens* | W | Wild | Rio Claro, Brazil | Giannotti, 1997 | 38.3 | 1 | 94 | 24.5 | 24.5 | 261 |
| WASP | *Polistes chinensis antennalis* | W | Wild | Ibaraki Prefecture, Japan | Miyano, 1980 | 37.8 | na | na | na | na | na |
| WASP | *Polistes gallicus* | W | Unknown | Italy | Pardi 1948 | 19.2 | ? | ? | ? | ? | ? |
| WASP | *Vespa ducalis* | W | Wild | Japan | Matsuura 1979 | 34.9 | ? | ? | ? | ? | ? |
| WASP | *Vespa crabro* | W | Wild | Japan | Matsuura 1979 | 16.6 | ? | ? | ? | ? | ? |
| WASP | *Vespula germanica* | W | Lab | USA | Hurd et al 2007 | 14.5 | 3 | 30 | na | 5.9 | 428 |
| WASP | *Vespula vulgaris* | W | Unknown | UK | Spradbery 1973 | 12.4 | ? | ? | ? | ? | ? |
| WASP | *Polybia occidentalis* | W | Wild | Guanacaste, Costa Rica | O'Donnell & Jeanne 1992 | 24.7 | na | na | na | na | unsure methods |
| WASP | *Polybia occidentalis* | W | Wild | Guanacaste, Costa Rica | O'Donnell & Jeanne 1992 | 20.1 | na | na | na | na | unsure methods |
| WASP | *Vespula consobrina* | W | Lab | Washington, USA | Akre et al 1982 | na | 3 | 31 | na | na | na |
| WASP | *Vespa velutina* | W | Lab | China | Dazhi and Yunzhen 1989 | na | 24 | 142 | na | na | na |
| WASP | *Polistes versicolor* | W | - | - | Gobbi, 1977 (in Giannotti, 2012) | na | 10.8 | 17.62 | ? | ? | ? |
